# Digestion and Dyspepsia

**Published:** 1887-02-19

**Authors:** 


### Digestion and Dyspepsia.

On Friday evening last, Dr. Tom Robinson, of Princes Street, Cavendish Square, gave a lecture on Dyspepsia at the Midwives' Institute, Buckingham Street, Strand. The attendance was large. The lecturer strongly cautioned nurses against trusting to patients in the matter of the selection of proper food. By the assistance of a large diagram, he explained the process of mastication, and the passage of food through the œsophagus into the stomach. Persons with uneven sets of teeth were better without them, as far as the purposes of mastication were concerned, as they were absolutely useless for biting food. The great and first step in digestion commenced in the mouth. The saliva turned all starch-matter into sugar. As an instance of this, he remarked that, if we took a piece of wholemeal bread into the mouth and chewed it, it became sweet. All forms of food should be well pulverised in the mouth. The œsophagus, through which the food next passed, was an organ which was seldom diseased. After passage into the stomach, the gastric juice and acid secretions so modified all the lean particles of food that they could be afterwards taken up by the bloodvessels. Innumerable gastric follicles were embedded in the lining of the stomach. The food was sent from side to side by the muscular coats of the stomach till it was well mixed, and then passing through the pylorus, became alkaline in the duodenum. The next process in digestion was the action of the bile. The difficulty of doctors in studying digestion had been very great. The common idea in the last century was that there was a "spirit" inside the body, which made the person sleep, walk, digest food, and so on! Some of the first experiments in regard to the process of digestion were made by an Italian physician, who put food into little bags, passed them down into the stomach of the person experimented upon, and pulled them up again for examination at intervals! The lecturer then detailed the experiments performed by Dr. Beaumont upon a man named St. Martin, who had the front wall of the stomach blown away, a circumstance which had led to the discovery of facts of the highest value in the study of the digestive process. Speaking of the value to medical science of operations upon the lower orders of animals, the lecturer said he thought the Government was wrong in checking doctors in this direction.

Dr. Robinson next dealt with the causes of dyspepsia, or indigestion, foremost among which was too much food. It was impossible to define exactly how much food was right to keep up the proper standard of health, for so many circumstances, such as the kind of food, the time, etc., had to be taken into consideration. As a rule, men ate too much food, and women too little, especially those in the upper classes. If a man ate too much food, the remedy was very simple—check the quantity. A frequent form of dyspepsia with ladies arose from their eating too little, especially in the cases of young ladies of from seventeen to twenty. They gave the doctors a deal of trouble. Their heads ached with the least exercise; they could not get up in the morning because they were always tired; they easily got short of breath, and were extremely irritable. It would generally be found that these young ladies were eating only as much as would keep a little pet dog alive. They took very little exercise, and, altogether, were going to the bad as far as their health was concerned. This form of dyspepsia was due simply to the fact that the stomach had not sufficient food. If the stomach were left without food, it went on working, wasted away, and got less and less muscular, till at last it refused to do its work at all. The remedies were: first, insist on outdoor exercise; and, secondly, insist on the patient taking food.

A common cause of indigestion was improper food. The more simply we lived, the better we were. His own food did not cost him ten shillings a week. With simple food we did our work infinitely better. It was not the workers in the world who gorged themselves. We ought not to make everything subservient to food. Unquestionably people did like eating; it was a pleasant process, but the thing was not to prolong it beyond what was necessary. Some people should avoid such things as pork, veal, new bread, stews, vegetables which grew underground, jugged hare, and eggs. He had a great antipathy to eggs as an article of diet when in town, owing to the state of decomposition in which they were frequently found in London. Another cause of indigestion was the improper eating of food. He considered that three meals a day were quite sufficient for ordinary, healthy people. A general mistake we made was in not having our breakfast good enough. Breakfasts were generally made of a light kind. They should be made much better. The stomach had had a long rest; it was vigorous, and able to digest and take food in the morning, while it could not so well do so at any other time of the day. The next meal should be in the middle of the day: we should call this dinner. He saw no reason why people should not dine in the middle of the day. The last meal should be taken about an hour before bed. It was a mistake to suppose it was harmful to take a meal before retiring to rest. In support of this view, the lecturer instanced the practice of animals lying down to rest immediately after taking their food. The question might be asked, "How am I to know when I have taken enough food?" It was really a question of common-sense. We should not go beyond the limit of satisfaction. Digestion was also influenced by cold. Excessive heat, too, begot indigestion. The digestive organs of the body did their work without our knowledge, will, or control, and in a perfectly painless manner.

In conclusion, Dr. Robinson dealt with the proper feeding of infants. There was nothing like the mother's own milk for the child. Babies did not do so well with foster-mothers. With regard to all artificial forms of food, he counselled nurses never to allow them till children cut their teeth. He thought harm was being done in the present day by the discovery of so many forms of peptonised food, because they digested the food outside the stomach instead of inside it, the consequence being that the stomach got weak. In all ordinary forms of indigestion he advised nurses to throw away their pepsin-powders.

On the motion of Mrs. Coleman, seconded by Mrs. Perry, a hearty vote of thanks was passed to Dr. Robinson for his very practical lecture.

